# The Great Debate With IBD Biosimilars: Pro: Biosimilars Should Be Routinely Used as a First Line Biologic and May Be Switched From Reference Biologics

**DOI:** 10.1093/crocol/otab015

**Published:** 2021-04-15

**Authors:** Jimmy K Limdi, Francis A Farraye

**Affiliations:** 1 Inflammatory Bowel Diseases Section, Department of Gastroenterology, The Pennine Acute Hospitals NHS Trust, Manchester Academic Health Sciences, University of Manchester, Manchester, UK; 2 Division of Gastroenterology and Hepatology, Inflammatory Bowel Disease Center, The Mayo Clinic, Jacksonville, Florida, USA

**Keywords:** inflammatory bowel disease, Crohn’s disease, ulcerative colitis, anti-TNF, reference product, biosimilars, CT-P13, infliximab-dyyb, extrapolation, switch

## Abstract

The relatively high cost of anti-TNF agents and looming or actual expiry of patents for several biologics have led to the development of “highly similar” versions of the “originator” drugs called “biosimilars.” The approval of biosimilars has been based on “extrapolation,” whereby approval is granted in licensed indications for the reference product without the need for clinical trials. We discuss efficacy and safety data in support of biosimilar use from prospective studies, switching from originator biologic, impact on immunogenicity, pharmaco-economic, and practical considerations for clinicians.

## INTRODUCTION

The last 2 decades have witnessed unprecedented advances in our understanding of the immunopathogenesis of the inflammatory bowel diseases (IBDs). Conventional management previously involved the use of broad-spectrum anti-inflammatory drugs such as amino-salicylates and corticosteroids or immunosuppressants such as thiopurines or methotrexate, often sequentially with the aim of relieving symptoms and preventing long-term complications.^1,2^ Meanwhile, evidence that uncontrolled inflammation may lead to progressive intestinal injury, irreversible bowel damage, and complications together with an improved understanding of disease severity and risk factors for disease progression has paved the way for the new paradigm in IBD management, referred to as “treating-to-target.” ^3,4^

Effectively, this implies the earlier use of our most effective therapies in “well-selected” patients (at higher risk of disease progression and related complications).^3,4^ The advent of anti-TNF therapy at the turn of the century, demonstrating efficacy in both induction and maintenance of remission, corticosteroid sparing effects, mucosal healing, and reduction in hospitalization and surgery, has redefined our perceptions around meaningful disease control.^5^ With over 2 decades of data and experience, anti-TNF therapies with their early onset of action, are highly effective for gastrointestinal and extraintestinal manifestations of disease, and remain the most widely used biologic class in the treatment of IBD.^6^ Biological therapies are, however, the major driver of healthcare expenditure for IBD patients.^7^ The relatively high cost of anti-TNF agents and looming or actual expiry of patents for several biologics have led to the development of “highly similar” versions of the “originator” drugs and are called “biosimilars.” ^8^ Market competition and resultant price reductions have translated into cost savings and improved access to anti-TNF biosimilars worldwide.^9^

## WHAT ARE BIOSIMILARS?

The World Health Organisation defines a biosimilar as a biotherapeutic product which is similar in terms of quality, safety and efficacy to an already licenced reference biotherapeutic product.^10^ Biosimilars are complex molecules made by living organisms and in view of their natural variability, biosimilars are not an exact copy of the originator/reference product (RP), as synthetic chemical drugs are. Biosimilars received European Medicines Agency’s (EMA) Committee approval in June 2013, with CT-P13 approved across all infliximab indications.^11^ CT-P13 was approved in 2016 by the Food and Drug Administration (FDA) with the name infliximab-dyyb.^12^ Several infliximab and adalimumab biosimilars have subsequently been approved.^11,12^

The approval of biosimilars has been based on “extrapolation,” whereby approval is granted in licensed indications for the RP without the need for clinical trials.^11,12^ Unsurprisingly, this has led to much debate and Skepticism from some in the clinical gastroenterology community. The FDA considered extrapolation valid, when the mechanism of action, pharmacokinetics, immunogenicity, and safety of an RP is similar in extrapolated and clinically tested indications.^12^

Binding and neutralization of TNF, both soluble (sTNF) and transmembrane (tmTNF) are common to all anti-TNF monoclonal antibodies. Notably, anti-TNFs also have other mechanisms of action, namely reverse signaling, induction of regulatory macrophages, and antibody-dependent cell cytotoxicity (ADCC).^8^ Whether ADCC has a role in mediating anti-TNF efficacy in IBD remains unclear.^13^ However, blockade of inflammatory cytokines, induction of apoptosis via reverse signaling, and induction of macrophages reducing T-cell proliferation were highly comparable for CT-P13 (infliximab biosimilar) and ABP 501 (adalimumab biosimilar) with their respective RPs.^13^ Notably, etanercept primarily blocks sTNF, but does not induce reverse signaling and consequently, neither RP etanercept nor its biosimilar are licensed in IBD.^13^ The basis for CT-P13 approval was data from 2 randomized controlled trials in rheumatoid arthritis and ankylosing spondylitis which showed equivalent outcomes between CT-P13 and infliximab RP and subsequently also that switching between RP-infliximab and CT-P13 had no detrimental effects on safety or efficacy.^14,15^ Efficacy, safety, immunogenicity, and data on switching and interchangeability are discussed below.

## WHAT ARE THE DATA FOR EFFICACY AND SAFETY OF ANTI-TNF BIOSIMILARS IN PROSPECTIVE IBD STUDIES?

A growing body of evidence supports the use of biosimilar infliximab in anti-TNF exposed and biologic-naive patients with IBD (Table 1). Jahnsen et al reported the first prospective observational study with CT-P13 in 2015.^16^ Seventy-eight patients received CT-P13 over 14 months of which 70% with Crohn’s disease (CD) and 75% with ulcerative colitis (UC) were biologic-naive. Clinical remission [Harvey–Bradshaw index (HBI) ≤4] was achieved by 79% of patients with CD and 56% patients with UC [Partial Mayo Score (pMayo) ≤2] by week 14. No unexpected adverse events were reported. In a prospective observational study from Hungary in 2017, of 353 IBD patients receiving CT-P13 [*n* = 209 (CD); *n* = 144 (UC)], clinical remission in CD was achieved in 49%, 53%, and 48% and correspondingly clinical response in 86%, 81%, and 65% of patients by week 14, 30, and 54, respectively.^17^ Clinical remission in UC was noted in 56%, 41%, and 43% of patients with clinical response noted in 74%, 66%, and 50% of patients by weeks 14, 30, and 54, respectively.^17^ Significantly lower response and remission rates were noted in anti-TNF exposed patients, with infusion reactions reported in 8.8%, infections in 9% and only one death.^17^

**Table 1.** Clinical efficacy of CT-P13 in IBD

Author	Year	Country of origin	Study design	IBD type	Patient number	Follow-up (wks)	Clinical response	Clinical remission	Adverse events (%)	IRR (%)
Jahnsen et al	2015	Norway	Observational	CD = 46 UC = 32	78	14	NA	CD: 79% at wk 14 UC: 56% at wk 14	NR NR	CD = 2.2 UC = 3.1
Gecse et al	2015	Hungary	Observational	CD = 209 UC = 144	353	14	CD: 86%, 81%, and 65% at wks 14, 30, and 54 UC: 74%, 66%, and 50% at wks 14, 30, and 54	CD: 49%, 53%, and 48% at wks 14, 30, and 54 UC: 56%, 41%, and 43% at wks 14, 30, and 54	5.7%	6.6%
Farkas et al	2016	Czech Republic and Hungary	Observational	UC	63	14	82.5% at wk 14	Steroid-free remission: 47.4%	ATI = 0.09%	NR
Fiorino et al	2017	Italy	Observational	CD = 373 UC = 307	680	32	Overall 30.9%	CD = 57% UC = 50%	12.1%	6.9%
Ye et al	2019	Sixteen countries	Double-blind, phase 3 noninferiority	CD	220	30	Clinical response	Clinical remission	64% (CT-P13–CT-P13) 62% (CT-P13–infliximab group) 69% (infliximab–infliximab) 73% (infliximab–CT-P13)	0.009%

Abbreviations: ATI, antibodies to infliximab; CD, Crohn’s disease; IBD, inflammatory bowel disease; IRR, infusion related reaction; NR, not reported; UC, ulcerative colitis.

The Italian PROSIT-BIO cohort is the largest prospective, multicenter observational study, reported clinical and endoscopic efficacy data in 680 consecutive patients [*n* = 373 (CD), *n* = 307 (UC)] and a mean follow-up of 32 weeks.^18^ Four-hundred patients were anti-TNF naive. Clinical remission [HBI ≤4 or Crohn’s disease activity index (CDAI) <150 for CD and pMayo <2 for UC] was achieved in 45.6% in the entire cohort, with deep remission noted in 57% of patients with CD and 50% of UC patients.^18^ In another multicenter study in 63 UC patients receiving CT-P13 from Hungary and the Czech Republic, clinical response and steroid-free remission was achieved in 82.5% and 47.4% of patients by week 14, respectively. Mucosal healing (Mayo E score 0 or 1) was noted in 47.6% of patients and complete mucosal healing (Mayo E score 0) in 27% patients.^19^ In a double-blind randomized parallel group phase 3 study in patients with moderate to severe CD, Ye et al compared the efficacy of CT-P13 to RP-infliximab in 220 patients across 16 countries.^20^ The primary outcome measure (CDAI; CDAI-70) response rate at week 6 was similar in both groups (69.4% with CT-P13 and 74.3% with RP-infliximab). CDAI-100 response was also similar in both groups (60.4% with CT-P13 and 64.2% with RP-infliximab). At week 30, CDAI-70 (76.6% with CT-P13 and 75.2% with RP-infliximab) and CDAI-100 response rates (72.1% with CT-P13 and 73.4% with RP-infliximab) were similar as were clinical remission (CDAI <150) rates (55% with CT-P13 and 56.9% with RP-infliximab).^20^ Finally, a retrospective multicenter study from India reported that biosimilar adalimumab was effective at inducing remission at week 8 in 46.9% patients with CD and 52.4% with UC and maintained at 1 year in by 32.7% CD and 33.3% UC patients, respectively.^21^

## SWITCHING FROM ORIGINATOR TO BIOSIMILAR AND INTERCHANGEABILITY: WHAT ARE THE DATA FOR NONMEDICAL SWITCHING?

A randomized noninferiority double-blind trial (the NOR-SWITCH study) examined the efficacy, safety, and immunogenicity of switching between RP-infliximab to CT-P13, in 482 patients across multiple indications, of which 32% had CD and 19% had UC.^22^ Patients were randomized in a 1:1 ratio to either continue RP-infliximab or switch to CT-P13. Across all indications, disease worsening occurred in 26% of patients in the RP-infliximab group and 30% of patients in the CT-P13 group. The frequency of adverse events was 10% in the RP-infliximab and 9% in the CT-P13 group.

The primary endpoint was designed to evaluate the occurrence of disease worsening in study participants across the disease groups. Notably, noninferiority was not shown for any of the diagnostic subgroups with the exception of spondyloarthritis.^22^ For CD, the confidence interval (CI) was close to inferiority for CT-P13. That said, NORSWITCH was not powered to demonstrate noninferiority within each diagnostic group considering the number of patients on stable treatment with infliximab originator eligible for inclusion, constraints on funds and the timeframe available, leading authors to caution against emphasizing treatment differences within each disease when interpreting these data.^22^

In the double-blind randomized parallel group phase 3 study in patients with moderate to severe CD by Ye et al discussed earlier, at week 30, patients were randomly assigned to a maintenance group [*n* = 56 (CT-P13) and *n* = 54 (RP-infliximab)] or a switch group [*n* = 55 (CT-P13 to RP-infliximab) and *n* = 55 (RP-infliximab to CT-P13)] with study drug administered in a double-blind manner.^20^ Efficacy was maintained from week 30 and was similar in all 4 treatment groups at week 54, with no clinically meaningful differences in immunogenicity.^20,21^

In the PROSIT-BIO cohort, 196/810 patients had previous exposure to biologics with 155 patients switched to CT-P13 after a mean 17 RP-infliximab infusions.^18^ Treatment failure was noted in 7.6% in the biologic-exposed group and 2% in those switched to CT-P13, whilst treatment efficacy was 71% (naive), 64% for biologic-exposed, and 82% for those switched to CT-P13, respectively. Infusion reactions occurred in 29% of biologic-exposed patients and 11.6% of those switched. Data on efficacy and safety of CT-P13 were similar to RP-infliximab, although no direct comparisons were performed. In another study from the Czech Republic that included 74 IBD patients who switched to biosimilar infliximab and 119 biologic-naive patients, remission rates at week 0 and 56 were similar at 72.2% and 77.8% and no difference in CRP or fecal calprotectin (FC) was noted at 56 weeks.^23^ There was no increase in immunogenicity in patients switched from originator to biosimilar infliximab. Adverse events were also compatible between both groups. In another study from the United Kingdom, 143 patients were switched from RP-infliximab to CT-P13. Drug survival was similar in the switched group and no differences in side effects reported.^24^ Likewise, in another study from Norway, where 143 patients were switched from RP-infliximab to biosimilar infliximab, 97% of patients remained on treatment for the entire 6-month follow-up period, with a very low rate of adverse events and no change in clinical or biochemical disease activity.^25^ In a large prospective observational study of 313 IBD patients switched from RP-infliximab to CT-P13, at 12 months, 68.2% of CD and 78.9% of UC patients were in remission.^26^ There was a low risk (2.7%) of anti-drug antibodies (ADAs) and serious adverse events occurred in 2.2%.^26^

Building on experience with wider adoption and reassurance from real-world data, a prospective cohort of 83 patients with >2-year follow-up found that 66% of IBD patients continued CT-P13 after switching from RP-infliximab, with no significant adverse events or significant change in ADAs or trough levels (TLs) through week 104.^27^ In a large prospective study from Japan, 372 of 523 patients switched from RP-infliximab to CT-P13 and followed for at least 4 months, demonstrated drug survival on biosimilar infliximab.^28^ Adverse events were noted in 20.3% of patients with infusion reaction being the most frequent in 9.4%.^28^ A systematic review of 11 observational studies reported outcomes in 829 patients treated with CT-P13. CT-P13 demonstrated an excellent efficacy and safety profile.^29^ Pooled rates of sustained clinical response among CD and UC after switching from infliximab to CT-P13 at 30–32 weeks were 0.85 (95% CI = 0.71–0.93) and 0.96 (95% CI = 0.58–1.00), respectively, and at 48–63 weeks were 0.75 (95% CI = 0.44–0.92) and 0.83 (95% CI = 0.19–0.99), respectively. Adverse events were rare (CD, 0.10, 95% CI = 0.02–0.31; UC, 0.22, 95% CI = 0.04–0.63).^29^

Lukas et al reported a recent experience of switching 93 patients from originator to biosimilar adalimumab and matched to 93 controls receiving originator adalimumab.^30^ No differences in either clinical or biochemical disease activity were noted between week 0 and 10 with no change in TLs either.^30^

Reassurance provided by “switch” data notwithstanding, concerns have been raised with “reverse-switching” that may arise from reimbursement policies.^31^ In a study from Hungary reporting a “reverse-switch” experience (biosimilar to originator infliximab) in 174 consecutive patients, there were no significant differences in the proportion of patients in clinical remission at week 8 before the switch (82.5% with CD and 82.9% with UC), at baseline (80.6% with CD and 81.6% with UC), at week 16 (77.5% with CD and 83.7% with UC), or at week 24 (CD 76.3% with CD and 84.9% with UC) (*P* = .60 among groups for patients with CD and *P* = .98 among groups for patients with UC).^31^ They observed no significant changes in clinical remission, TLs, and antidrug antibodies levels in this patient population.

A recent systemic review evaluated nonmedical switching of originator infliximab to an infliximab biosimilar in patients with IBD. It included 49 studies, including 5 reports from 3 randomized controlled trials (1 publication; 4 abstracts) and 44 reports from 40 observational trials and 1 case series (21 publications; 23 abstracts), representing a total of 44 distinct studies.^32^ The authors concluded that nonmedical switching of originator infliximab to infliximab biosimilar was safe with no efficacy, safety, or immunogenicity concerns. However, there were only 3 small number of randomized controlled studies included in this analysis with a predominance of observational cohort studies. Table 2 provides a summary of switch studies.

**Table 2.** Summary of studies on switching from RP-infliximab to CT-P13

Author	Year	Country of origin	Study design	IBD type	Patient number	Follow-up (wks)	Efficacy outcomes	Immunogenicity
Jorgensen et al	2017	Norway	Randomized noninferiority double blind	CD = 155 UC = 93	234	52	Disease worsening at wk 52: CT-P13 30% vs IFX 26%	New ADA in 8% CT-P13 switch group and 7% in RP-IFX group
Kolar et al	2017	Czech Republic	Observational	CD = 56 UC = 18	74	56	Clinical remission 72.2% at wk 0 vs 77.8% at wk 56	ADA 9.5% at wk 0 and 6.0% in switched patients
Rasanskaite et al	2017	UK	Observational	CD = 118 UC = 23 IBDU = 2	143	52	No difference in mean albumin, CRP, and CBC. Improvement in IBD-control score after switch	No significant difference in ADA or TL after switch [Pre: 73.2 ± 83.0 AU/mL; Post: 06.1 ± 102.0 AU/mL (*P* = .24)]
Fiorino et al	2017	Italy	Observational	CD = 53 UC = 44	97	6.1 ± 2.2 mo	Treatment persistence: 94.5% at 8 wks, 90.8% at 16 wks, and 78.9% at 24 wks	NA
Buer et al	2017	Norway	Observational	CD = 99 UC = 44	143	24	Sustained remission: CD = 87% baseline and 81% at 6 months UC: 88% at baseline and 95% at 6 months	Three patients developed ADA after switch
Bergqvist et al	2018	Sweden	Observational	CD = 195 UC = 118	313	52	Change in clinical disease activity at 2, 6, and 12 months after switch	2.7% developed ADA
Smits et al	2019	Netherlands	Observational	CD = 57 UC = 24 IBDU = 2	83	16	HBI ≤4 in CD and SCCAI ≤3 in UC	TL: 3.5 µg/mL at baseline, 4.2 µg/mL at wk 16 (*P* = .01) Two patients developed ADA after switch
Ye et al	2019	Multicenter (16 countries)	Randomized, multicentre, double-blind, phase 3 noninferiority study	CD	55 IFX-CT-P13	6	CDAI-70 response rates similar for CT-P13 [77 (69.4%, 95% CI 59.9 to 77.8) of 111] and infliximab [81 (74.3%, 95% CI 65.1 to 82.2) of 109]; difference [−4.9% (95% CI −16.9 to 7.3)]	NA
Lukas et al	2020	Czech Republic	Observational	CD = 80 UC = 13	93	10	No difference in the disease activity in SWITCH and ORIGINATOR cohorts between wks 0 and 10	Adalimumab levels stable after switch

Abbreviations: ADA, anti-drug antibody; CBC, complete blood count; CD, Crohn’s disease; CI, confidence interval; CRP, C-reactive protein; HBI, Harvey–Bradshaw index; IBD, inflammatory bowel disease; IBDU, inflammatory bowel disease unclassified; SCCAI, simple clinical colitis activity index; RP, reference product; TL, trough level; UC, ulcerative colitis.

## DOES THE USE OF BIOSIMILAR ANTI-TNF IMPACT ON IMMUNOGENICITY AND PHARMACOKINETICS?

Anti-TNF therapies are immunogenic and are associated with loss of response.^33^ Up to 30% of patients have a primary nonresponse and up to 50% will develop a secondary loss of response to anti-TNFs.^33^ The risk of attrition can make each successive therapy less effective, implying that the first biologic is often most likely to be the most effective. Furthermore, patients developing antibodies to the originator infliximab have a 2-fold increased risk of acute infusion reactions and a 6-fold increased risk of serious acute infusion reactions.^34^ Data on immunogenicity and infusion reactions with biosimilars appear reassuringly comparable to originator anti-TNF. In the PLANETAS and PLANETRA studies in patients with AS and RA, respectively, the rate of infusion reactions was 3.9% and 4.9% for AS and 6.6% and 8.3% for RA with CT-P13 and RP-infliximab, respectively.^14,15^ In the open-label PLANETRA extension study, patients who had completed the 54-week, randomized, parallel group study received CT-P13 (3 mg/kg) intravenously every 8 weeks from weeks 62 to 102.^35^ Among 302 of 455 patients who completed the PLANETRA study 158 had received CT-P13 (maintenance group) and 144 RP (switch group). Response rates at week 102 for maintenance versus switch groups, respectively, were comparable across groups. The proportion of patients with antidrug antibodies (week 102: 40.3% vs 44.8%, respectively) and treatment-emergent adverse events were similar (53.5% and 53.8%, respectively) in the 2 groups during the extension study out to 2 years.^35^

Both studies also showed comparable efficacy, immunogenicity and safety, 1 year after switching from RP-infliximab to biosimilar CT-P13. In a nationwide Hungarian study, mean TLs did not differ between UC and CD patients, except that early (week 6) TLs were higher in CD than in UC patients (18.4 vs 6.2 µg/mL; *P* < .001).^36^ Notably, patients who had prior anti-TNF exposure had lower TLs than anti-TNF naive patients but the differences were not significant.^36^ In a subsequent study from the same group, higher ADA positivity was noted in anti-TNF exposed patients (24.1% in naive and 44.4% in anti-TNF exposed) with early ADA formation prevented by concomitant thiopurine therapy in line with previous data from RP-infliximab use.^17,33,37^

The issue of cross-immunogenicity was examined by Ben-Horin et al.^38^ In this in vitro study, antibodies to originator infliximab from previous exposure were cross-reactive with CT-P13, suggesting similar immunogenicity shared by these agents.^38^ A central European study reported on the immunogenicity of CT-P13 in 384 patients from Hungary and the Czech Republic.^39^ Mean CT-P13 TLs were 20.1, 14.7, and 5 µg/mL at weeks 2, 6, and 14, respectively. Correspondingly, ADA positivity rates were 8.7% 19.3%, and 28% at weeks 0, 14, and 30, respectively.^39^ Infusion reactions were noted in 7.3% of patients being most frequent with the second and third infusion and were comparable between RP-infliximab and CT-P13. Subsequent studies have continued to build confidence with comparable immunogenicity. Kolar et al reporting from a Czech cohort found no difference in ADA positivity (9.5% vs 6.0%, *P* = .54) after switching and week 56 post switch from RP-infliximab.^23^

The SECURE study reported a prospective evaluation of drug levels after switching from RP-infliximab to CT-P13 in 120 consecutive patients with IBD.^40^ The primary endpoint was serum concentrations of infliximab 16 weeks after switching, assessed separately in patients with UC and those with CD in the per-protocol population. The geometric mean ratio of serum infliximab concentrations at week 16 (CT-P13) compared with those at baseline (originator) was 110.1% (90% CI 96.0–126.3) in patients with UC and 107.6% (97.4–118.8) in those with CD,^40^ thus demonstrating noninferiority of CT-P13 with RP-infliximab. In the double-blind randomized parallel group phase 3 study in patients with moderate to severe CD, comparing CT-P13 with RP-infliximab, the rate of infusion reactions was similar in both groups (CT-P13 7.2% and 8.3£ with RP-infliximab) at week 30.^20^ Finally, in the 78-week SB2 phase 3 transition trial in patients with rheumatoid arthritis, assessing efficacy, safety, and immunogenicity with a switch from infliximab to SB2, infliximab/infliximab, and SB2/SB2 groups at 78 weeks, among patients who were negative for antidrug antibodies (ADAs) up to week 54, newly developed ADAs were reported in 14.6%, 14.9%, and 14.1% of the infliximab/SB2, infliximab/infliximab, and SB2/SB2, respectively.^41^ Taken together, data for immunogenicity with biosimilars are reassuring and comparable to originator anti-TNF.

## EVOLVING PERCEPTIONS AMONG CLINICIANS AND PATIENTS

The complex process of development of biosimilars may result in slight changes in the molecular construction even without any modifications in the molecular construct which ensures “biosimilarity.” ^42^ It is, however, also clear that even the originator product will have seen some changes over time from the very processes that involve the protein structure of biosimilar production. A growing body of evidence continues to reassure clinicians with respect to efficacy and safety of biosimilar anti-TNFs in bio-naive patients and also patients previously exposed to originator anti-TNF products as discussed extensively above. These changing perceptions were well captured by 2 surveys conducted by ECCO in 2013 and 2015. In the first survey, over 60% of clinicians expressed little or no confidence with biosimilar prescribing and expressed significant concerns with validity of extrapolating across indications, immunogenicity, switching from originator to biosimilar anti-TNF and with automatic substitution.^43^ In the follow-up survey in 2015, however, fewer (20%) expressed lack of confidence with biosimilars, 44% (from 6% in 2013) were more confident with switching from originator anti-TNF and fewer clinicians (33% compared to 76%) were against extrapolation across indications.^44^ Immunogenicity remained a concern (69%) although cost savings and their translation into better patient care was welcomed by 92% of respondents.^44^ Automatic substitution (when a pharmacist or healthcare provider may replace a branded drug prescribed by a physician with a generic version without consulting the prescribing specialist) remains a concern with 90% clinicians in the follow-up survey having strong reservations against this. Although commonplace with synthetic drugs, automatic substitution is not supported by international recommendations and there are no data from the IBD literature to support this practice.^8,45^

Scientific credibility notwithstanding, patient awareness and knowledge underpins ultimate acceptance of treatment and avoidance of nocebo effects (worsening of symptoms triggered by negative feeling toward an new intervention).^46^ A European Federation of Crohn’s and Ulcerative Colitis Association survey noted that only 38% of 1181 patients surveyed had even heard of biosimilars, with concerns regarding safety and efficacy expressed by 46.5% and 38.6% patients, respectively.^47^ In a study of 125 patients with IBD and rheumatological conditions switched from RP-infliximab to biosimilar infliximab, an overall 12.8% nocebo response was noted, despite no statistically significant changes in effectiveness and safety across indications over 9 months.^48^ Good communication, positive framing and informed consent, have been shown to mitigate against unwanted nocebo effects.^8,45,49^ A recent single-center prospective observational study measured health-related quality of life (HRQoL), in 54 IBD patients receiving maintenance IFX therapy after switching from originator to biosimilar IFX.^50^ HRQoL was measured, using the 15D health-related quality of life instrument (15D) and the Inflammatory Bowel Disease Questionnaire (IBDQ). CDAI or pMayo, and FC were assessed for evaluation of disease activity, at switch and 3 and 12 months after switching. No statistically significant changes were observed in 15D, CDAI, pMayo, and FC during 1-year follow-up. IBDQ scores were higher (*P* = .018) in CD, 3 months after switching and costs of biosimilar IFX were one-third that of originator infliximab.^50^

## PHARMACO-ECONOMICS

It has to be conceded that the main driver behind production of biosimilars was reduced cost which can drive market competition, budget sustainability and improved patient access to biological therapies, rewarded by reinvestment of these savings into improved and earlier access to treatment, and improved quality of care for patients with IBD.^51^ Indeed, data from the CALM trial did show that “tight control” using biomarker directed treatment was cost effective versus the standard clinical management arm over 2 and 5 years, with cost effectiveness improving over time.^52^ The UK National Health Service (NHS) reported £324 million in cost savings in the 2017–2018 financial year by switching to biosimilars or generics for 10 medicines of which £100 million were saved using infliximab biosimilars alone.^53^ A budget impact analysis from lower and middle income European countries reported 8 million EUR in saving with the 25% price reduction in biosimilars even if only new IBD patients were treated with biosimilar infliximab over 3 years and 16.9 million EUR saved if 80% of IBD patients received CT-P13, allowing switching from originator, allowing between 722 and 1530 patients to be treated with biosimilar infliximab.^54^ Likewise, a budget impact analysis from higher income European countries assuming price reductions of 10%, 20%, and 30%, respectively, reported expected cost savings increasing from 11.9 million EUR to 35.8 million EUR in CD and between 5.1 and 15.4 million EUR in UC, enabling an additional 1219–4701 additional patients be treated with biosimilar infliximab.^55^

## CONCLUSION

The approval of biosimilars through abbreviated licensing pathways was largely driven by burgeoning costs and looming expiry of licensed biologics. Extrapolation of data across all indications without the need for clinical trials as the basis of approval was met with appropriate Skepticism. However, a growing body of evidence has provided reassuring data with respect to effectiveness and safety of biosimilar anti-TNFs in bio-naive patients, patients previously exposed to originator anti-TNFs and with nonmedical switching to biosimilar anti-TNF agents in stable patients. Patient education regarding the use of biosimilars especially when a patient is switched from RP to biosimilar is crucial in order to decrease nocebo rates. Current evidence for patients with IBD does not, however, support multiple switching or automatic substitution at pharmacy level, nor negate the need for careful on-going pharmaco-vigilance. Nonetheless, significant cost reductions will hopefully trigger a virtuous self-perpetuating cycle with wider access to early and effective therapy in appropriately selected patients. In a world of increasing inequity with health care, biosimilars are a welcome addition to our therapeutic armamentarium, helping to achieve meaningful equipoise with our most altruistic patient centered objectives.

## Data Availability

This is a review article and as such no new data were created or analyzed.

## Notes

Author Contribution: J.K.L. conducted the literature search and wrote the manuscript. J.K.L. and F.A.F. critically reviewed the manuscript for intellectual content. All authors approved the final version.

Funding: J.K.L. have received speaker/consultancy fees from Abbvie, Arena Pharma, Galapagos, Janssen, Takeda and Pfizer.
